# Comprehensive Analysis of Steel Slag as Aggregate for Road Construction: Experimental Testing and Environmental Impact Assessment

**DOI:** 10.3390/ma14133587

**Published:** 2021-06-28

**Authors:** Marina Díaz-Piloneta, Marta Terrados-Cristos, Jose Valeriano Álvarez-Cabal, Eliseo Vergara-González

**Affiliations:** Project Engineering Department, University of Oviedo, 33004 Oviedo, Spain; marta.terrados@api.uniovi.es (M.T.-C.); valer@uniovi.es (J.V.Á.-C.); vergaraeliseo@uniovi.es (E.V.-G.)

**Keywords:** steel slag, road materials, life cycle assessment, experimental testing, recycled materials, circular economy

## Abstract

Blast Oxygen Furnace (BOF) slag represents one of the largest waste fractions from steelmaking. Therefore, slag valorisation technologies are of high importance regarding the use of slag as a secondary resource, both in the steel sector and in other sectors, such as the construction or cement industries. The main issue regarding the use of BOF slag is its volumetric instability in the presence of water; this hampers its use in sectors and requires a stabilisation pre-treatment. These treatments are also cost-inefficient and cause other environmental issues. This paper analyses the use of untreated BOF slag from a technical and environmental point of view, suggesting it as an alternative to natural aggregates in road surface layers and asphalt pavements. A comprehensive analysis of the requirements to be met by raw materials used in asphalt mixes was performed, and a pilot test was carried out with two different mixtures: one mix with limestone as coarse aggregate and another with 15% BOF slag. Furthermore, the global warming impacts derived from each mix with different aggregates were measured by Life Cycle Analysis (LCA), and a transport sensitivity analysis was also performed. The results show how the utilization of BOF slag as coarse aggregate in road construction improves the technical performance of asphalt mixtures (Marshall Quotient 4.9 vs. 6.6). Moreover, the introduction of BOF slag into the asphalt mix as a coarse aggregate, instead of limestone, causes a carbon emissions reduction rate of more than 14%.

## 1. Introduction

Steel slag is the main source of solid waste in the steel industry [1]. It is obtained either by melting scrap with a high electric current in an electric arc furnace (EAF) or by processing hot melted metal, scrap and fluxes with lime in a basis oxygen furnace (BOF). Around 70% of the world’s steel production depends on the blast furnace process (integral route), as the availability of scrap limits electric arc furnace production to 30% of global demand [2]. In the BOF process, the amount of BOF slag produced ranges between 0.1 and 0.2 tonnes per tonne of steel produced [3,4]. In 2017, around 1200 million tonnes of steel were produced by the integral route and around 480 million tonnes were produced by the electric route [5]. These production data translate into the generation of between 120 and 240 million tonnes of BOF slag in just one year. Furthermore, steel production has increased considerably in recent years, and annual consumption is expected to grow 3.3%, which could reach an output of 2.4 billion tonnes by 2025 [6]. Therefore, increasing production of steel slag is becoming a serious disposal problem.

Given this, slag valorisation technologies are of high importance in the use of slag as secondary resource, both for the steel sector and for other sectors, such as the construction or cement industries. This fact has prompted scientists and engineers to work on novel solutions based on more eco-friendly industrial concepts, mainly in the field of construction [7,8]. Roads are the most dominant forms of transport infrastructure in Europe, and a major contributor to the European economy. Almost three quarters of inland freight transport in Europe is carried out by road [9]. In addition, a considerable amount of new and rehabilitated roads is built every year; for instance, more than 250 Mt of asphalt mixes were produced in 2019 in Europe alone [10]. This road network involves not only the consumption of raw materials but also energy use (electricity and fuels), generating a significant environmental impact. 

The road sector is one of the main contributors to global warming [11]. The greenhouse gas (GHG) emissions per meter per year associated with the construction of road infrastructure has been estimated at 14.7 kg CO_2eq_. Several steps contribute to the production of GHG, but the material extraction and production stage has been identified as the main contributor to total carbon emissions and energy consumption [12]. Hence, the choice of materials impacts local pollution and environmental degradation [13].

Aggregates are the main material used in road pavements. The composition of average asphalt concrete is approximately 90% aggregates, 3% filler, 2% additives and 5% binder [14]. The constructing of a single km of new road requires the consumption of 30,000 tonnes of aggregate [15]. This fact notwithstanding, large quantities of BOF slag can meet the needs of aggregates for road construction. The use of waste and by-products is one of the most promising techniques for sustainable roads, as it provides a double benefit [16]. On the one hand, it reduces the extraction and production of raw materials, reducing the consumption of water, electricity and diesel, as well as the generation of other diffuse emissions such as noise and dust. On the other hand, it avoids the disposal of waste in landfills, extending their useful life and reducing emissions.

The main problem regarding the use of BOF slag is its volumetric instability in the presence of water [17]. This is the result of it having high percentages of free lime and magnesium oxides that have not reacted with the silicate structures. When slag hydrates, its volume increases, and this swelling can lead to structural problems. This hydration expansion of BOF slag limits its engineering applications.

For this reason, several authors have investigated methods for slag stabilisation. The ageing treatment is a passive, low cost, simple and effective method [18]. It basically consists of naturally storing the material until it stabilises over time. However, this process takes a long time, usually longer than a year, which affects the progress of a given project. Moreover, this pre-treatment can also involve environmental problems due to the land occupation caused by the disposal of the material [19]. For this reason, this method is sometimes accelerated with steam, high pressure water jets or by tumbling and vibrating the slag. Another stabilization process is the use of acidic compounds that react with steel slag and remove free lime [20,21]. Covering the surface of BOF slag is another treatment that has been applied by Chen et al. [22]. The fully hydrated slag was immersed in silicon resin for 1 h and then cured in an oven at 60 °C for 24 h. The process needs to be repeated for three cycles. The study evaluated slag’s viability as an aggregate for use in asphalt mixes. However, although the slag achieved the required levels of stability, the impact on the mechanical and thermal performances of the mixture is unclear.

All these treatment techniques reduce the free-lime content in slag and allow its reuse, but they are time-consuming and cost-inefficient. If slag needs to be pre-treated before it can be used, it is very difficult for it to compete with natural aggregates for road construction from an economic point of view. In addition, these pre-treatments involve the consumption of other raw materials (energy, fuels, etc.), which also involves environmental impacts. Therefore, it is necessary to encourage the use of untreated slag as much as possible.

There are many studies on the use of BOF slag for road construction, but they use treated slag or remain at a laboratory level, as reflected in the reviews conducted by Kambole et al. [23] and Kumar and Varma [23,24]. Another example is the work carried out by Dayioglu et al. [25]. They evaluated BOF ageing slag that was coated with asphalt cement. The slags were also mixed with water treatment residuals for their use in highway base and coarse layers. In this regard, some authors suggested a possible stabilisation of the slag by adhesion with the asphalt binder [26]. The results showed that, if the slag was encapsulated by hydrated products, the contact between BOF slag and rainwater decreased because of the coating that bitumen provides. Therefore, the binder protected the material from swelling by water; thus, it could be used even when untreated. In addition, one potential hazard regarding the use of BOF slag is an increase in the chemical elements released into the environment because of its contact with rainwater (especially chromium and vanadium) [27]. However, when it is used as an aggregate that is encapsulated by a binder, this problem can be controlled because of the coating that bitumen provides [28]. 

Laboratory analysis shows that the inclusion of BOF slag in an asphalt mix may enhance skid resistance [19], mechanical properties [29] and rutting resistance [30]. Another study shows how mixing limestone with steel slag as a coarse aggregate can provide an asphalt mixture with high resistance to plastic deformation and good resistance to fatigue failure [31]. Amir et al. [32] analysed the long-term fatigue behaviour of asphalt mixtures containing BOF slag subjected to long-term ageing. The results showed that the addition of aged EAF slag improved fatigue life. Kong et al. [33] investigated volumetric and mechanical properties to evaluate the influence of the geometric characteristics of BOF slag coarse aggregates. They concluded that BOF slag has higher angularity than natural aggregates, and can enhance the properties of moisture damage and rutting resistance in the asphalt mixture.

In comparison to laboratory studies, field research provides a better understanding of long-term performance and mechanical properties. However, there is a lack of studies in a real environment. Xie et al. [34] evaluated the deleterious potential and heating characteristics of BOF slag based on laboratory and in-place investigation during large-scale reutilisation. In a constrained space, BOF was stewed at about 600 °C and 4 kPa for nearly 8 h. They used treated BOF aggregates for paving the surface layer of a road. A series of tests were carried out to check the road performance after construction, and the results met the requirement of Chinese norm JTG F40-2004. The results of another investigation into BOF slag used as aggregate in stone mastic asphalt showed that it performs as well as the conventional asphalt pavement [35]. Due to the instability and expansibility of steel slag, they performed an activation test with water until an expansion of about 1% was achieved. Subsequently, they paved an old two-way, four-lane highway. After two years of service, the slag asphalt section showed no signs of rutting, cracking or stripping.

In most of the works analysed, the use of BOF slag for road construction has remained largely at the research level; when applied in real cases, it is usually in relation to a pre-treatment. There is an urgent need to develop and demonstrate such solutions extensively in real-life environments, and it is also essential that the slag used is untreated in order to promote market acceptance. In addition, it is important to carry out an analysis of the environmental gains from the use of BOF slag compared to natural aggregates. Although there are some studies that analyse the environmental impact of the use of slag on roads, most focus on the arbitrary substitution rates supported by the literature [36,37]. The results show an environmental gain with the use of BOF slag instead of natural aggregates, but they are not based on proven technical studies on a real scale.

Therefore, the novelty of this study lies in two main facts. On one hand, it carries out full-scale research with untreated BOF slag and does not remain at the laboratory level. On the other hand, this paper examines the technical feasibility of used waste in road construction with real data, and analyses its environmental impacts via a Life Cycle Analysis (LCA). The quantities and materials used are based on a real test with proven technical performance to ensure the correct use of BOF slag on road construction.

The objectives of the present work are as follows:

- Laboratory analysis to ascertain the suitability of BOF slag as a coarse aggregate in wearing courses;
- Comparison between the one-year performance of pavements with BOF slag as coarse aggregate and conventional pavements with natural aggregates in a real environment;
- The difference between the characteristics of natural aggregates and BOF slag and pavement mix designs are discussed;
- An assessment of the environmental sustainability of the use of BOF slag in road pavements is carried out by a life cycle analysis.

In Section 2 we present the materials and methods used to carry out this work. In Section 3, we detail the test we developed and discuss the different results. Finally, the main conclusions and future lines of inquiry are presented in Section 4.

## 2. Materials and Methods

**Figure 1:**
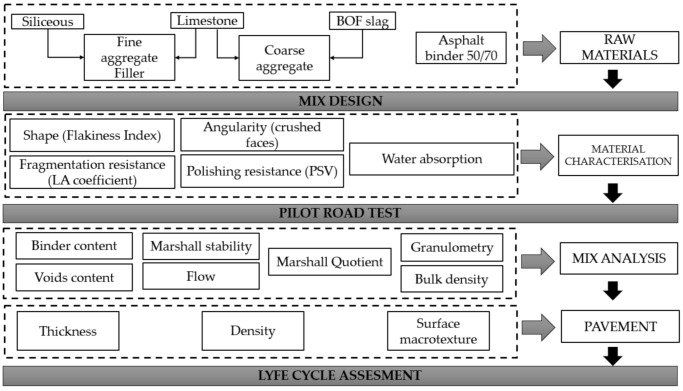
Experimental design program (LA: Los Angeles coefficient; PSV: Polished Stone Value).

An experimental design program for this research project was developed to achieve the presented objectives, as shown in Figure 1. First, a comprehensive analysis of the requirements to be met by raw materials used in the asphalt mix was performed. Subsequently, the pilot test was carried out, with analyses of the mix and the pilot pavement; finally, the impacts derived from each of the mixes were measured by life cycle analyses. 

### 2.1. Materials

Asphalt mixtures are divided into hot and cold types depending on their manufacture and application. Hot mix asphalts require heating to reduce viscosity. They are a combination of coarse (>2 mm) and fine aggregates (2/0.063 mm), filler (<0.063 mm) and binder [38]. Specifically, asphalt concrete mixes (AC) use bitumen as a binder [39]. The aggregates act as the structural skeleton of the pavements, whereas the hydrocarbon ligand homogeneously covers all the materials. This is the most widely mix used in roads, and it needs to be of a high quality; that is why we have selected for our research [40].

Two scenarios, with two asphalt mixtures and different types of aggregate, were examined in this study. Limestone and siliceous aggregates constituted the baseline scenario (AC-base). The second one (AC-BOF) used BOF slag as coarse aggregate instead of limestone (Table 1). The slag replacement content was approximately 15% of the aggregate’s weight. The asphalt concrete mixtures were designed according to Spanish standards using conventional 50/70 penetration-grade bitumen [41]. The particle size distribution was defined following the limits established by the international regulations for pavement design (EN 933-2).

The BOF slag used as a second type of coarse aggregate in this research was provided by a Spanish steel production company. Limestone and siliceous aggregates are easily available, and they were obtained from nearby quarries. The coarse aggregate characteristics must meet different requirements depending on their final use.

The use of BOF slag as a coarse aggregate in asphalt concrete was evaluated by comparing the test results obtained with the limits established in different countries and other natural aggregates (ballast, limestone and siliceous). In order to analyse these limits, the following tests were carried out:

- Water absorption (EN 1097-6);
- Angularity (for particles partially or completely crushed, EN 933-5);
- Shape (flakiness index test, EN 933-3);
- Fragmentation resistance (Los Angeles coefficient, EN 1097-2);
- Resistance to polishing of the coarse aggregate in road layers (Polished Stone Value, EN 1097-8).

### 2.2. Road Test

Pavement maintenance depends on the country and the budget. When a pavement surface is damaged, the damaged section can be removed and resurfaced with AC of the same thickness [42]. The test carried out in this study followed this principle of regular maintenance. A damaged section of surface pavement of approximately 150 m^2^ (40 m long) was removed, and then two sections of BOF slag AC (20 m) and conventional AC (20 m) were paved, with a maximum nominal size of 16 mm. The mix used was AC16 surf 50/70 D.

The road section is located in Gijón, Spain. The specific requirements of aggregates and asphalt mix depend on the category of the road and the average daily traffic of heavy vehicles (ADHTV) (T [43]. The most restrictive categories are T00 and T0 (between 4000 and 2000 heavy vehicles per day. In this case, the original road was designed primarily for small and medium sized vehicles (T3-T4) (200–25 heavy vehicles/day).

To determine the practical AC mix design, the optimal amount of asphalt binder to be used (EN 12697-39), the density (EN 12697-6 and EN 12697-5), the air voids (EN 12697-8) and the stability value and Marshall performance (EN 12697-34) were studied. The ratio stability (kN) to flow (mm), called Marshall Quotient (kN/mm), was also calculated.

The one-year performance of the road was evaluated using various on-site tests: measurement of the depth of the pavement’s surface macrotexture using the volumetric method (circle of sand), according to EN 13036-1; determination of its apparent density by the hydrostatic method, according to EN 12697-6; and its thickness by the destructive method, according to EN 12697-36. The extraction of the cores was carried out according to EN 12697-27:2001. 

### 2.3. Environmental Impact Assessment

The results of several studies on asphalt pavements have shown that the stages with the highest environmental impacts are those corresponding to the extraction and processing of raw materials [44,45,46]. Therefore, reintroducing recycled materials into the process helps to conserve resources and reduce waste [47,48]. The environmental analysis of BOF slag as raw material in asphalt concrete manufacturing was carried out by the LCA methodology. 

Environmental performance was evaluated by comparing two scenarios: a first standard mix of conventional asphalt (AC-base) with natural aggregates (limestone, basalt and siliceous), and a second one in which a percentage of coarse aggregates were substituted (AC-BOF). Comparative LCA was used in other studies to test the environmental feasibility of using slag asphalt [49]. The considered service-life was 20 years. The methodology used is based on ISO 14040 [50]. The data collected for the life cycle were analysed with SimaPro v8 software (2016, SimaPro, Amersfoort, The Netherlands). According with the 2030 Framework and the Paris agreement, the European Union has the objective of reducing 55% of GHG before 2030. Then, global warming potential (GWP) was selected as an environmental indicator. Although other impacts such as the use of resources could be considered, these correspond to abundant materials in the area, and the effect of their use is very limited.

**Figure 2:**
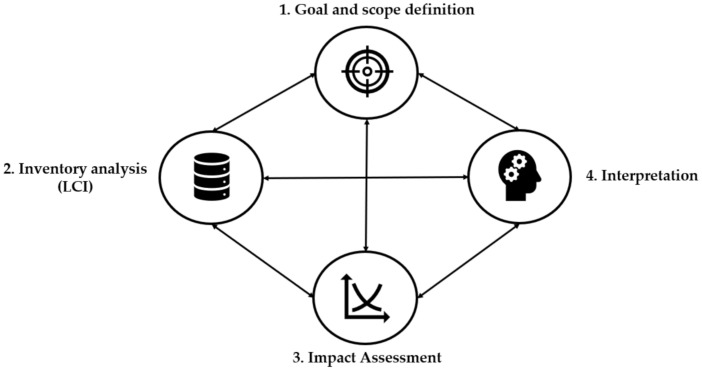
Main phases of a Life Cycle Analysis study.

As can be seen in Figure 2, LCA methodology can be synthesised in four main phases. The first one is focused on defining the objectives, the functional unit and the scope (functional limits of the system). The life cycle inventory identifies and collects the data. Inputs and outputs associated with the processes within the system boundaries are identified and quantified. During the third phase, the evaluation of the significant potential environmental impacts is carried out using the data from the inventory phase. The last phase includes the interpretation of the results, as well as the eventual compiling of conclusions and recommendations for the improvement of the environmental performance of the system. 

#### 2.3.1. Goal and Scope Definition

Our main objective is to study the environmental impacts of the use of BOF slag as a substitute for raw material in an asphalt mix. This is achieved by comparing two scenarios. The first one, AC-Base, involves traditional asphalt mixes with different natural aggregates (basalt, siliceous aggregates and limestone). The second scenario, AC-BOF, involves natural aggregates that have been partially replaced by BOF slag. The slag replacement content was 15% of the aggregate’s weight. This will prevent the accumulation of the waste in landfills, as well as the extraction of new raw materials. In addition, there are other secondary objectives:

- Encouraging the use of recycled materials to improve the environmental sustainability of asphalt pavements;
- Enhancing the circular economy, thus avoiding waste that will be reintroduced in a productive cycle;
- Avoiding emissions from resource extraction.

**Figure 3:**
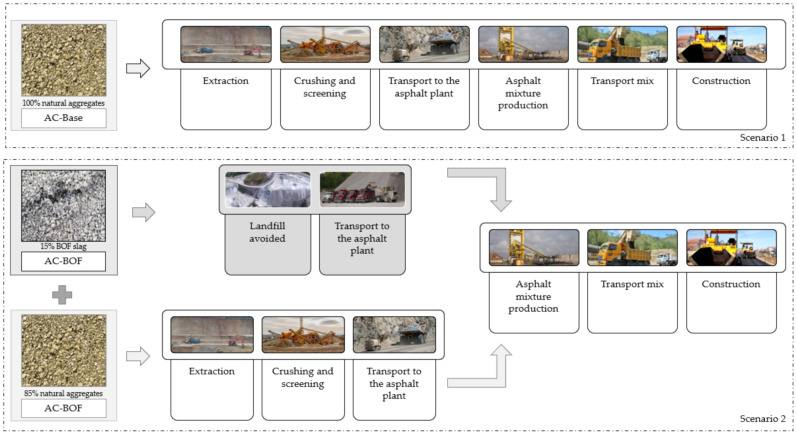
Description of the system boundaries in both scenarios (AC-Base: mix with natural aggregates; AC-BOF: mix with BOF slag).

The functional unit is “1 m^2^ of pavement for small and medium sized vehicles”. Regarding system boundaries, the material production, transport and construction stages were taken into account (Figure 3). Therefore, the approach encompasses from “cradle”, where raw materials are extracted, put into production and used, to “gate”, regarding use, maintenance and end of life stage. Those elements with very low influences on the environmental impact were excluded, as well as those without control during their design. The results will have a non-generalist character, as they are directly conditioned by the hypotheses and limitations.

The life cycle in this study started from the stage of raw materials’ production, which included aggregates and bitumen. The environmental impacts mainly come from mineral extraction, asphalt refinement and distribution of the aggregates to the asphalt-mixing plant. The distance between the production site of the aggregates, both BOF slag and natural aggregates, and the asphalt plant is estimated at 20 km on average. These are common data used by default in road project budgets in the region. However, the transportation phase is an important life cycle stage for aggregates, and it largely affects total energy consumption and emissions. Depending on local availability of materials, road transportation may vary accordingly.

Natural aggregates should be crushed and screened for use in the asphalt mix; meanwhile, the bitumen would be heated. In the case of slag, these treatments were carried out by the producer. Furthermore, the slag used did not require any pre-treatment for its stabilisation. The asphalt mixture was produced and transported to the pavement construction site. The environmental impacts in this stage are mainly caused by the burning of fossil fuels. Regarding construction stage, the environmental impacts involve the same processes for both pavements, so it is assumed that there are no significant differences.

#### 2.3.2. Inventory Analysis (LCI)

In order to be able to simulate the product through its inputs and outputs, the system was defined in terms of its functional unit. Therefore, through the inventory analysis (LCI), energy resources and material flow within the road’s construction, maintenance and end-of-life process are defined and quantified into categories of environmental metrics. In both scenarios, the background data needed were extracted from the Ecoinvent 3.2 database [51]. Table 2 shows the system inputs. The quantities are given for one square metre (approximately 133 kg). The quantities were estimated on the basis of the measurements and tonnes of asphalt used in the test (20 t).

The extraction process of natural aggregates included crushing and screening. In the case of limestone (“Limestone, crushed, for mill {GLO} (GLOBAL)”), the infrastructure data in Ecoinvent were estimated based on a normalized infrastructure with an annual production capacity of about 145,000 tonnes of product per year, which is very similar to the quarry used for the road test. Regarding basalt (“Basalt {GLO}”), the module included a mining step and a subsequent beneficiation step comprising crushing and classification. Finally, siliceous aggregates (“silica sand {GLO}”) also included crushing and milling processes. 

Slag utilization in the road construction industry means that dumping on landfills is avoided. This is an avoided impact, which was taken into account as an environmental credit in the AC-BOF scenario. Therefore, BOF slag impacts were reflected in the transport process to the asphalt plant. Data from Ecoinvent dataset were applied in order to evaluate the related environmental burdens and the energy requirements associated with this process. For the transport of both aggregates, the service of “1tkm freight transport in a lorry of the size class 7.5–16 metric tonnes gross vehicle weight, EURO6” was considered. The transport datasets refer to the entire transport life cycle, i.e., to the construction, operation, maintenance and end of life of vehicle and road infrastructures. Fuel consumption and emissions were calculated for average European journeys and load factors, and were not representative of a specific transport scenario.

Regarding bitumen, the life cycle inventory for the production of 1 tonne of bitumen, from oil extraction to storage in the refinery (Table 3), was considered for each scenario with help from the European Bitumen Association [52]. Since the quantities involved are very similar in both scenarios, and small amounts are required, the transport of bitumen from the refinery to the asphalt plant was neglected. 

#### 2.3.3. Impact Assessment

The objective of this stage is to analyse the data collected during the previous inventory phase and to assess the effects on the environment, specifically in relation to the carbon footprint. There are different assessment methods and, in this case, the IPCC (Intergovernmental Panel on Climate Change) methodology was used [53]. It contains the climate change factors of IPCC with a timeframe of 100 years. This method is focused on the direct (except CH_4_) global warming potential of air emissions. They are:

- Not including indirect formation of dinitrogen monoxide from nitrogen emissions;
- Not accounting for radiative forcing due to emissions of NOx, water, sulphate, etc. in the lower stratosphere + upper troposphere;
- Not considering the range of indirect effects given by IPCC;
- Not including indirect effects of CO emissions.

## 3. Results and Discussion

### 3.1. Technical Behaviour of BOF Slag as Coarse Aggregate for Road Construction

According to the methodology described in Section 2.1, the test results of the technical specifications associated with the Spanish requirements [41] for coarse aggregates in their wearing use are summarized in Table 4.

These parameters reflect the aggregates’ performance and, therefore, the asphalt pavement. The abrasion value of the aggregates or Los Angeles coefficient (LA) represents the mass loss of the aggregate in the abrasion process. This property denotes the hardness or toughness of aggregates and can show the durability of asphalt pavements [19]. Aggregates with low abrasion value have high mechanical strength and abrasion resistance. If the aggregates used exhibit inadequate toughness and abrasion resistance, the asphalt pavement may show construction and performance problems [55]. The BOF slag analysed shows a Los Angeles coefficient of 14, which is lower than limestone value (LA = 25) and other natural aggregates such as basalt (LA = 14.9) and siliceous aggregates (LA = 20). Thus, BOF slag has good toughness and abrasive properties, and it meets the requirements established for the most demanding pavements (T00 and T0).

The polishing property of aggregates is a key property that denotes surface microtexture, which is an important aspect of skid resistance and driving safety [56]. The Polished Stone Value (PSV) measures an aggregate’s resistance to a polishing action that is similar to the movement of a vehicle’s tyres. [57]. The PSV of BOF slag (PSV = 56) is the highest value of the aggregates analysed. Only when PSV is more than 50 and LA value is less than 20 can the aggregates be used in anti-slip layers [19]. 

A crushed or broken particle is defined in EN 933-5 as a particle whose surface is crushed or broken by more than 50%. Measuring the crushed faces of an aggregate is one way of measuring its angularity. Angularity is a description of the edge sharpness of an aggregate’s particles. There is a fixed limit for the percentage of fracture faces, with the purpose of maximizing the shear strength asphalt mixtures [33]. Therefore, the standards are demanding and usually set at a value of 100% for the strongest pavements. BOF slag complies with requirements and no differences with the rest of the aggregates analysed can be seen.

The physical shape of coarse aggregate is an important property in the performance of bituminous mixes. Flakiness index (FI) is one of the prominent shape criteria that governs behaviour and performance of aggregates in asphalt pavements [58]. The existence of flaky aggregates in bituminous mixes is undesirable and a dangerous phenomenon because of their tendency to break under wheel load, either during compaction in the construction stage or in the service life of the pavement [59]. A higher FI indicates a larger number of elongated particles. BOF slag has the lowest value, 8.1; therefore, it offers a higher rutting resistance. 

The water absorption of aggregates is an indication of the number of inter-connected voids of a size that will allow free water to move into them upon soaking. Aggregates with absorption values over 5% should be avoided in hot mix asphalt [23]. According to Spanish standards [41], this value is only limited to wearing courses subjected to frequent frost and winter maintenance. If the water absorption value (EN 1097-6) is higher than 1%, the magnesium sulphate test value (EN 1367-2) should be lower than fifteen percent (MS < 15%). BOF slag exceeds the permitted value for the water absorption test but does not exceed the threshold for the magnesium sulphate test (MS = 0.26%).

**Figure 4:**
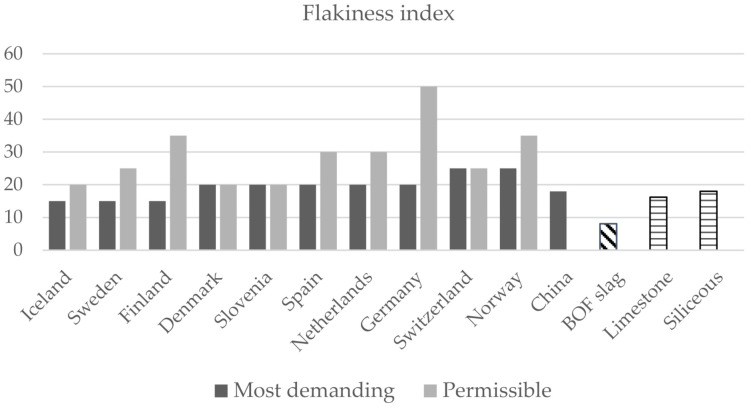
International comparison requirements for aggregates in road construction: Flakiness index.

**Figure 5:**
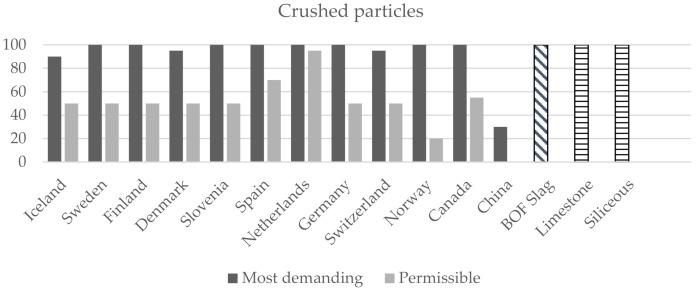
International comparison requirements for aggregates in road construction: Crushed particles.

**Figure 6:**
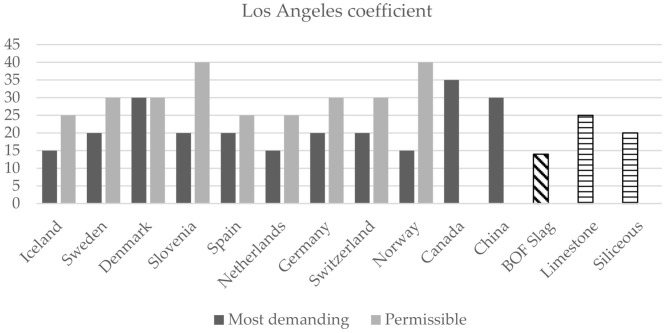
International comparison requirements for aggregates in road construction: Los Angeles coefficient.

On the other hand, the requirements established in other countries for coarse aggregates in asphalt mixes have been analysed. The limiting parameters most commonly used are shown in Figure 4, Figure 5 and Figure 6 [40,60]. In this case, the road category has not been considered, and only the most restrictive (most demanding) and permissible values set in the different countries are shown.

As can be seen, BOF slag as a coarse aggregate also meets the more restrictive limits of other countries. The flakiness index is the reference test for determining the shape of aggregates. Iceland, Sweden and Finland are the countries that set the most demanding value (<15) and, according to the analyses, carried out, the flakiness index for BOF slag is 8.1; therefore, the requirements are met. The Los Angeles abrasion value was much lower for BOF slag than it was for the selected natural aggregates, which confirms the superior frictional resistance properties of the slag. BOF slag also complies with the most restrictive values for the Los Angeles abrasion coefficient (<15 in Iceland, Norway and Netherlands) and crushed particles which, in most cases, should be 100%.

### 3.2. Road Test Performance

**Figure 7:**
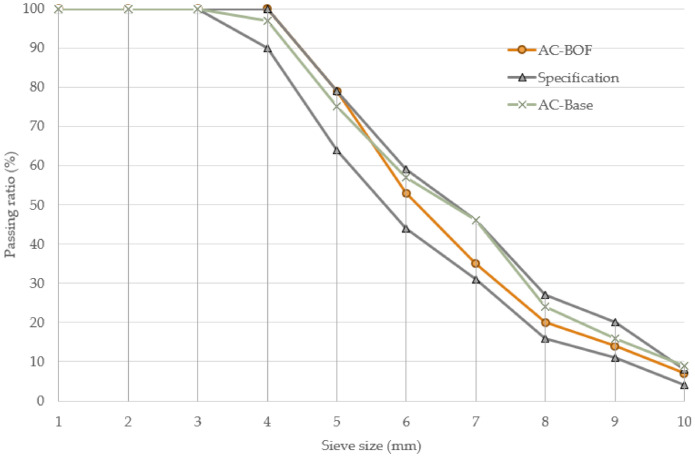
Particle size distribution gradations curves for conventional mix (AC-Base) and BOF slag mix (AC-BOF).

Regarding mix requirements, Figure 7 shows the aggregate gradation curves for AC mix designs according to EN 12697-2. The black line is the limit curve in accordance with the standard requirements. The studied mixes present a continuous and well-graded particle size distribution, and they are within the range according to the specifications. There are no significant differences between the two mixtures. The minimum binder content for wearing courses, according to the standard, is 4.5%. The optimum binder content found for both mixtures was 4.96% for AC-Base and 5.27% for AC-BOF. This suggests that the introduction of BOF slag showed little effect on the asphalt content. 

The properties of the analysed mix for each sample are shown in Table 5. These properties are essential for verifying the success of the asphalt’s design and preparation, as well as to establish the air void content of the mixtures, which is a key characteristic of a bituminous mix. The Marshall test provides important values for the performance of an asphalt mix. This test looks for the highest stability and lowest flowability. Marshall stability is the ability of a mix to resist shoving and rutting under traffic. Flowability is the ability of the asphalt concrete to resist settlement and gradual movement without cracking [61]. The results indicate that the Marshall stability and bulk density increases in mixtures with BOF slag. Finally, the Marshall Quotient can be used as a measure of a material’s resistance to permanent deformation while in service. A higher value in the Marshall Quotient indicates a stiffer and more resistant mixture [62]. 

In traditional asphalt mixture gradation designs, air voids in the asphalt mixture are the most important indexes of volumetric properties; they can also affect the performance of an asphalt pavement. Several studies have been conducted to determine the effect of air voids on the performance characteristics of asphalt mixtures, including fatigue cracking and rutting [63]. These reports suggest that the road service life decreases by approximately one year for every 1% increase in the air void content. In this case, the mix with natural aggregates has a higher void content (4.5%) than the mix with slag (4.1%).

As far as the finished road is concerned, the surface macrotexture of the AC-Base is 0.6 ± 0.1 mm, and it is 0.7 ± 0.1 mm in AC-BOF. Thickness is similar in both cases, being 55 and 57 mm, respectively. Under these conditions, after nearly 1 year of service, the BOF slag test road shows excellent performance without rutting, cracking or spalling, all of which cause asphalt pavement to be damaged early on. 

### 3.3. Life Cycle Analysis

**Figure 8:**
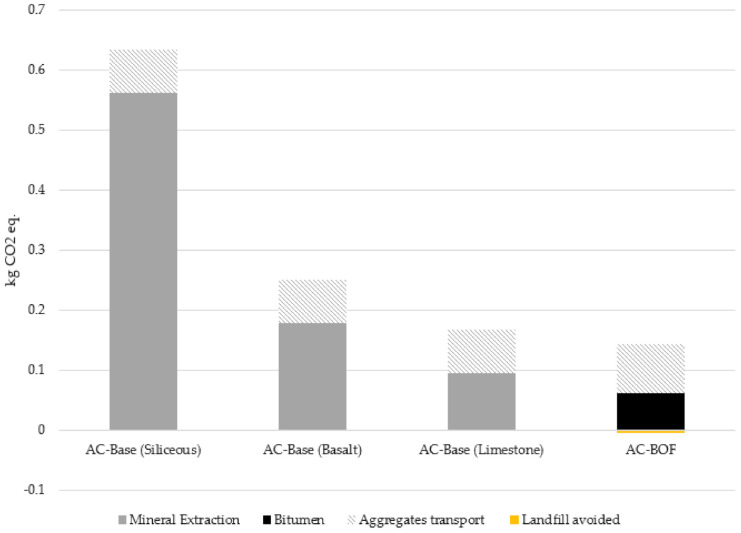
Comparative global warming potential for different scenarios.

Based on the inventory data and assumptions previously described, global warming potential was calculated (cradle to gate). This impact has been analysed by comparing the use of different natural aggregates (limestone, basalt, siliceous aggregates) used in conventional asphalt mixes and BOF slag as a substitute waste (Figure 8).

Compared to the considered natural aggregates, BOF slag contributed greatly to reducing CO_2eq_ emissions. In comparison to the baseline scenario for the real road test (AC-Base Limestone), the introduction of BOF slag into the asphalt mix as a coarse aggregate, instead of limestone, involves a carbon emissions reduction rate of more than 14%. The case of siliceous aggregates is more interesting, with an approximate 78% reduction in emissions. This result implies that, for every square metre of BOF slag-based mix, almost 0.5 kg of CO_2eq_ could be saved. 

It is important to highlight the positive impact of avoiding filling landfills with slag. The results show a small impact compared to the other phases considered, and the analysis is not able to reflect the problems involved in slag disposal. The high generation rates mean that storing slag is a real environmental problem. Landfills are often full, so even avoiding the disposal of a relatively small amount of this waste and giving it a second life is a crucial achievement and an imperative need. 

**Figure 9:**
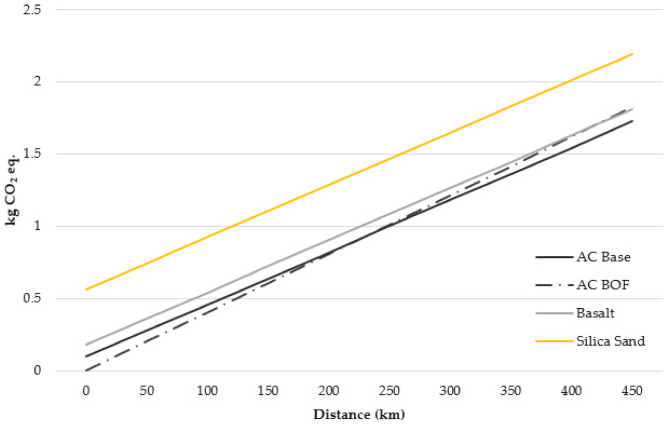
Sensitivity analysis for transport distances for different aggregates.

The phase with the greatest impact is mineral extraction and, in the case of BOF slag, the transport of waste to the asphalt plant. At present, the availability of BOF slag mainly depends on metallurgical manufactures; therefore, its utilization is considerably affected by cost-efficient transportation distances. In addition, BOF slag has a high density (approximately 3 t/m^3^), so transport is a decisive factor in its reuse. The sensitivity analysis of transport distances comparing different aggregates is shown in Figure 9. The scenario considered sets a distance limit of 500 km for aggregate transport. A preliminary analysis shows interesting results when considering that each compared aggregate and BOF slag will be freighted for an equal distance. In this setting, and regarding the carbon footprint, the use of siliceous aggregates cannot be considered. In the case of limestone, the BOF slag scenario still exhibits benefits for delivery distances up to 200 km. Finally, a rejection of BOF slag in favour of basalt, at least in terms of delivery distance, should only be considered if the former has to travel 400 km or more. 

These findings notwithstanding, the distances from the aggregate quarry to the asphalt plant, as well as the distances involved in BOF slag production, differ depending on particular conditions. Therefore, a more interesting comparison would be to consider the carbon footprint of different transport distances for each aggregate. If the transport distance is not the same, the use of BOF slag may not be worthwhile from an environmental point of view. 

For example, if there is a limestone quarry 50 km away but BOF slag’s is 100 km away, limestone would be more cost-effective when considering CO_2_ emissions. Similarly, if basalt’s transport is longer than 200 km and BOF slag’s is 250 km, the former will have less impact. While the difference between limestone and basalt is very small compared to slag, the difference with siliceous aggregates is very large. Thus, even if siliceous aggregates have a short transport distance (such as 50 km), BOF slag would still have a lower carbon footprint in journeys up to almost 200 km.

**Figure 10:**
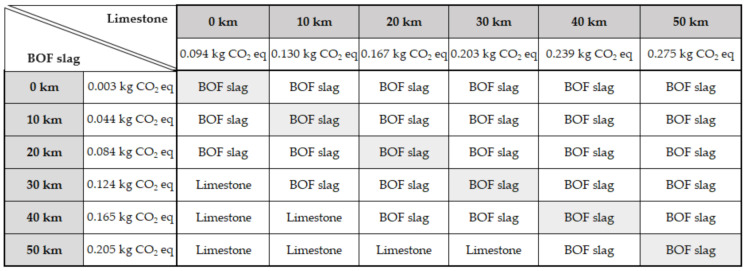
Decision tool comparing limestone and BOF slag as aggregates used in asphalt mixes.

Figure 10 represents a decision support tool for BOF slag and limestone. The CO_2_ emissions were calculated considering the transport of 15% of the substituted aggregates. Analysing distances of up to 50 km between limestone production and slag production, both in relation to the asphalt plant, shows that, in more than 80% of the possible scenarios, the use of BOF slag pays off from an environmental point of view.

Thus, the curves presented in the Figure 9 are an interesting decision support tool for road project managers. With this tool, they can choose the most suitable aggregate for an asphalt mix based on distance and carbon footprint.

## 4. Conclusions 

In this study a comparative comprehensive analysis of the use of BOF slag as coarse aggregate in road surface layers was performed. As the discussed results show, the utilization of BOF slag improves the technical and environmental performance of asphalt mixtures compared to the natural aggregates we tested (limestone, basalt, siliceous aggregates). 

From a technical point of view:

- A series of tests to check the physical and mechanical properties of coarse aggregates used in road construction have been carried out (Los Angeles abrasion, Flakiness Index, water absorption, Polished Stone Value, etc.);
- A real study has been carried out comparing two different mixes by replacing 15% of the coarse aggregates with BOF slag;
- After nearly 1.5 years of service, the BOF slag road test showed excellent performance without rutting, cracking or spalling, all of which cause asphalt pavements to be damaged early on;
- It can be concluded that BOF slag has better properties than conventional natural aggregates, and can therefore improve road performance.

From an environmental point of view:

- The carbon footprint of different mixtures has been assessed and a transport sensitivity analysis has been carried out;
- The main limitation of the environmental study lies in the quality of the primary data used. In this way, the results could be improved if the data were gathered directly at the test site;
- Taking into account this limitation, the main result is to be able to obtain the delivery distance limit at which there is no longer an environmental gain from using BOF slag. This enables decision making by road project managers from a sustainable point of view.

The road test has been in use for more than a year, but promising results are expected in the long term. In this respect, future work is planned to extend the scope of the life cycle analysis from cradle to grave, including maintenance, use and end-of-life stages, when more data on pavement performance will be available. As recommendations for further works, special attention should be paid to the composition of leachates, specifically the chromium and vanadium values. In addition, it would be interesting to analyse the impact of a higher percentage of BOF slag substitution on the amount of asphalt, as well as the effects of rheology and temperature on transport costs. Other possible environmental impacts should also be considered.

## Figures and Tables

**Table 1:** Aggregates and particle size used in mix design (AC-Base: mix with natural aggregates; AC-BOF: mix with BOF slag).

Particle Size (mm)	AC-Base (Natural Aggregates)	AC-BOF (BOF Slag)
0/4	Limestone	Limestone
0/4	Siliceous	Siliceous
4/12	Siliceous	Siliceous
10/20	Limestone	Steel slag

**Table 2:** The construction materials used in the conventional (AC-Base) and alternative (AC-BOF) scenarios.

Input	Unit	AC-Base	AC-BOF
Natural coarse aggregate (limestone, basalt, siliceous)	kg	126.72	107.35
Bitumen	kg	6.61	7.03
BOF slag	kg	0	18.95
Distance	km	20	20

**Table 3:** Life Cycle Inventory to produce 1 ton of bitumen.

Process	Variable	Unit	Parameter
Raw material	Crude oil	kg	1000
Consumption of energy resources	Natural gas	kg	25
	Crude oil	kg	14
Consumption of non-energy resources	Water	L	342
Emission to air	CO_2_	g	136,797
	SO_2_	g	813
	NO_x_	g	881
	CO	g	58
	CH_4_	g	392
	NMVOC	g	361
	Particles	g	80

**Table 4:** Requirements of coarse aggregates to be used in wearing coarse.

Parameter	Standard	Heavy Traffic Category							Limestone	BOF Slag	Siliceous [54]	Basalt [35]
		T00	T0	T1	T2	T31	T32	T4				
Los Angeles coefficient (LA)	EN 1097-2	≤20	≤20	≤20	≤20	≤25	≤25	≤25	25	14	20	14.9
Polished Stone Value (PSV)	EN 1097-8	≥56	≥56	≥56	≥50	≥50	≥44	≥44	NA	56	52	49
Crushed particles (%mass)	EN 933-5	100	100	100	100	≥90	≥90	≥70	100	100	100	NA
Flakiness index (FI)	EN 933-3	≤20	≤25	≤25	≤25	≤30	≤30	≤30	16.2	8.1	18	NA
Water absorption (%)	EN 1097-6	<1 *							0.3	2.4	1.5	0.7

* If the aggregates are not compliant with the water absorption test, it is necessary that the magnesium sulphate test (EN-1367-2) is <15%. NA= Non data available.

**Table 5:** Summary of principal properties of tested mixtures.

Parameter	Standard	AC-Base	AC-BOF
Bulk density (kg/m^3^)	EN 12697-6	2390	2445
Maximum density of the mixture (kg/m^3^)	EN 12697-5	2449	2550
Void content (%)	EN 12697-8	4.5	4.1
Voids Filled with Bitumen (%)	EN 12697-8	72.4	75.4
Marshall stability (kN)	EN 12697-34	13.6	15.20
Flow (mm)	EN 12697-34	2.78	2.32
Marshall Quotient (kN/mm)	EN 12697-34	4.9	6.6

## Data Availability

All the data is available within the manuscript.
